# Advanced magnetic resonance imaging of chronic whiplash patients: a clinical practice-based feasibility study

**DOI:** 10.1186/s12998-022-00410-y

**Published:** 2022-01-07

**Authors:** Lars Uhrenholt, Lau Brix, Thea Overgaard Wichmann, Michael Pedersen, Steffen Ringgaard, Tue Secher Jensen

**Affiliations:** 1 Department of Forensic Medicine, Aarhus University, Palle Juul-Jensens Blvd. 99, 8200 Aarhus N, Denmark; 2 Nortvig & Uhrenholt Kiropraktisk Klinik, 8200 Aarhus, Denmark; 3 Department of Radiology, Diagnostic Centre, University Research Clinic for Innovative Patient Pathways, Silkeborg Regional Hospital, Falkevej 1-3, 8600 Silkeborg, Denmark; 4 Department of Procurement and Biomedical Engineering, Region Midt, Aarhus N, Denmark; 5 Department of Neurosurgery, Aarhus University Hospital, Aarhus, Denmark; 6 Comparative Medicine Lab, Department of Clinical Medicine, Aarhus University, Aarhus, Denmark; 7 MR Research Centre, Clinical Medicine, Aarhus University, Aarhus, Denmark; 8 Chiropractic Knowledge Hub, Odense, Denmark; 9 Department of Sport Science and Clinical Biomechanics, University of Southern Denmark, Odense, Denmark

**Keywords:** Whiplash injury, Magnetic resonance imaging, Cerebrospinal fluid, Neck pain, Imaging study

## Abstract

**Background:**

Whiplash injury is common following road traffic crashes affecting millions worldwide, with up to 50% of the injured developing chronic symptoms and 15% having a reduced working capability due to ongoing disability. Many of these patients receive treatment in primary care settings based upon clinical and diagnostic imaging findings. Despite the identification of different types of injuries in the whiplash patients, clinically significant relationships between injuries and chronic symptoms remains to be fully established. This study investigated the feasibility of magnetic resonance imaging (MRI) techniques including quantitative diffusion weighted imaging and measurements of cerebrospinal fluid (CSF) flow as novel non-invasive biomarkers in a population of healthy volunteers and chronic whiplash patients recruited from a chiropractic clinic for the purpose of improving our understanding of whiplash injury.

**Methods:**

Twenty chronic whiplash patients and 18 healthy age- and gender matched control subjects were included [mean age ± SD (sex ratio; females/males), case group: 37.8 years ± 9.1 (1.22), control group: 35.1 years ± 9.2 (1.25)]. Data was collected from May 2019 to July 2020. Data from questionnaires pertaining to the car crash, acute and current symptoms were retrieved and findings from clinical examination and MRI including morphologic, diffusion weighted and phase-contrast images were recorded. The apparent diffusion coefficient and fractional anisotropy were calculated, and measurement and analysis of CSF flow was conducted. Statistical analyses included Fisher’s exact test, Mann Whitney U test and analysis of variance between groups.

**Results:**

The studied population was described in detail using readily available clinical tools. No statistically significant differences were found between the groups on MRI.

**Conclusions:**

This study did not show that MRI‐based measures of morphology, spinal cord and nerve root diffusion or cerebrospinal fluid flow are sensitive biomarkers to distinguish between chronic whiplash patients and healthy controls. The detailed description of the chronic whiplash patients using readily available clinical tools may be of great relevance to the clinician. In the context of feasibility, clinical practice-based advanced imaging studies with a technical setup similar to the presented can be expected to have a high likelihood of successful completion.

## Background

Whiplash is a common injury following road traffic crashes affecting several million people worldwide each year. A significant proportion, up to 50%, develop chronic/long-term symptoms [1–3] and approximately 15% of those initially injured suffer from reduced working capability due to ongoing disability [3]. Chronic whiplash patients often complain of neck pain, headaches and pain in the cervico-thoracic region of the spine. In addition, the majority of these patients suffer from varying degrees of non-painful neurological symptoms, *e.g.* paresthesia, dizziness, fatigue, memory deficit and other cognitive symptoms. The risk of chronic symptoms can be predicted based on early stratification in clinical settings and the prognosis for full recovery is poor if symptoms persist beyond one year [3, 4]. In most cases of acute whiplash injury the causal inferences are readily made in the clinical setting, i.e. acute symptoms (temporality) following a rear-impact collision (relevant trauma) and consistency of clinical findings (complaints, signs and symptoms).

The etiology of chronic symptoms following whiplash injury is however more challenging although a prerequisite is an acute whiplash injury and continued symptomatology over a period of at least six months. Although different types of injuries in chronic whiplash patients have been identified and debated, including for example injuries to the cervical facet joints [5–8], ligamentous instability in the craniocervical region [9–11], cervical spine muscle fat infiltration [12, 13] and tonsillar ectopia [14], there is currently no evidence to support an association between identifiable structural/somatic injuries and chronic symptoms following whiplash injury [15]. Similarly, pathophysiological findings have been observed, e.g. lowered pain pressure threshold, altered balance and visual control, and hypersensitivity of the central nervous system [16–20]. Psychological studies have correlated anxiety, catastrophizing, post-traumatic stress disorder symptoms and pre-impact health to the prognosis following whiplash injury [21, 22]. However, there is no strong evidence for an association between these factors and the prognosis after whiplash injury [23].

The question remains whether currently undisclosed pathoanatomical and/or pathophysiological conditions may explain the development of chronic symptoms following whiplash injury. While clinical evaluation may reveal areas of injury and/or poor function, the lack of scientific evidence of specific injuries, e.g. pathognomonic lesions, from imaging studies following whiplash trauma is challenging to clinical practice. The clinicians still need to base their clinical decision strategies primarily on clinical findings generally without support of objective documentation from radiological imaging.

A recent post-mortem diffusion weighted imaging (DWI)-based magnetic resonance imaging (MRI) study introduced three-dimensional reconstruction of the spinal cord and the cervical spine nerve roots, demonstrating a positive correlation between morphological MRI and DWI [24]. Hence, this method may be valuable in evaluating patients suffering from symptoms related to the cervical spine nerve roots and/or spinal cord. With the exception of one recent study [16], the cervical spinal cord or nerve roots in whiplash-injured patients have not been examined using DWI. In addition, the movement and function of the cerebrospinal fluid (CSF) that surrounds the cervical spinal cord may play a role in the clinical course of chronicity. The concept of CSF flow has a central role in alternative medicine, e.g. cranial osteopathy and craniosacral therapy through the so-called “primary respiratory mechanism”. In recent years, there has been increased attention to characterize the CSF flow in neurological diseases, e.g. Chiari malformation and cervical myelopathy [25, 26]. In this context, phase-contrast MRI (PC-MRI) can be utilized to characterize the CSF flow dynamics within the spinal subarachnoid space [27, 28]. However, the role of CSF flow measurements in chronic whiplash patients remains to be elucidated [29, 30].

In this study, we aimed to investigate the feasibility of MRI techniques including quantitative DWI and measurements of CSF flow as novel non-invasive biomarkers in a population of healthy volunteers and chronic whiplash patients recruited from a chiropractic clinic.

## Methods

### Patient population

Chronic whiplash patients and healthy controls were eligible for inclusion in this case–control study. Whiplash patients (cases) were defined according to the preceding trauma, the consequent persistent clinical symptoms and the clinical examination. The cases had been involved in a motor vehicle collision and suffered from ongoing (chronic) symptoms since onset for a minimum of six months, including at least daily neck pain and stiffness. Cases were recruited from the participating chiropractic clinic. Inclusion required musculoskeletal signs and symptoms, including reduced cervical spine range of motion, neck pain reproduction by muscle, ligament or joint palpation and/or loading through cervical spine compression and/or distraction, thereby classifying the subject as a Quebec Task Force on Whiplash Associated Disorders grade 2 (QTF WAD grade 2) according to international standards [31]. Cases were excluded when previous serious head-/neck injury, cervical spine fracture/dislocation, neurological symptoms to the extremities, spinal cord and/or neurologic diseases, claustrophobia and/or competing general illnesses were documented. The control subjects should be in good health with no current neck pain or neck pain episodes within the past 12 months that required treatment, and they were excluded when previous serious head-/neck injury, cervical spine fracture/dislocation, neurological symptoms to the extremities, spinal cord and/or neurologic diseases, claustrophobia and/or competing general illnesses were documented.

### Case history and questionnaires

All participants answered an electronic questionnaire containing information regarding their gender, age, occupation and general health. The cases responded to questions regarding the traffic crash, e.g. seating position, direction of impact, use of seatbelt and deployment of airbag. They reported on both acute and current symptoms, including a visual analogue scale (VAS) from 0 to 100 mm for acute and current neck pain and headache. Two questionnaires were completed; the Neck Disability Index (NDI) and the Copenhagen Neck Function Disability Scale (CNFDS). The control subjects similarly reported on current symptoms including a VAS for current neck pain and headache, and they completed the NDI and CNFDS if relevant. The NDI was evaluated based on the following scale; NDI 0–4 = no disability, 5–14 = mild, 15–24 = moderate, 25–34 = severe and 35–50 = complete [32]. Similarly, the CNFDS was evaluated based on the following scale; CNFDS 0 = no disability, 1–3 = minimal disability, 4–8 = mild disability, 9–14 = mild to moderate disability, 15–20 = moderate disability, 21–26 = moderate to severe disability and 27–30 = severe disability [33].

### Clinical evaluation

Clinical data were retrieved from all subjects including a neurological screening of the cranial nerves and peripheral nerves to the upper extremities, orthopedic testing of the cervical spine, active cervical range of motion (ACROM) in 6 rotational directions of the three-dimensional Cartesian coordinate system (repeated three times using a clinical goniometer), reporting of upper limb tension tests (ULTT) for the median, radial and ulnar nerves, reporting of tender points (TeP) by quantitative algometry using a Wagner Instruments^©^ algometer by Method 1; six points repeated three times each (posterior part of C2, C5 and m. tibialis anterior bilaterally), and Method 2; full 18 points algometry using the American College of Rheumatology TePs using a pain threshold of 4 kg/cm^2^ [34], and testing for hypermobility according to the Beighton scale.

### Acquisition of magnetic resonance imaging

The MRI examinations were carried out on a Siemens Skyra 3T MRI system (Software Release E11a, Siemens Healthcare GmbH, Erlangen, Germany) using a head posterior (20 channel) and a thoracic spine (24 channel) coil. The MRI cervical imaging protocol included a sagittal 2D T2-weighted Fast Spin Echo sequence (DIXON fat suppression), a sagittal 3D T2-weighted SPACE sequence, a sagittal 2D T1-weighted Fast Spin Echo sequence, a non-gated transversal 2D readout-segmented EPI (Echo-planar Imaging) diffusion weighted sequence with 6 diffusion directions and b-values of 0 and 600 s/mm^2^, and a 2D transversal retrospective gated phase-contrast CSF flow imaging sequence (see Table 1 for details). The head/neck region of the patients were immobilized as much as possible prior to scanning using comfort cushions in order to reduce any excess motion during scanning. No further motion correction was added to the images after acquisition.

**Table 1:** MRI sequence parameters

Sequence	TR	TE	Slice thickness	Flip angle	Slice Spacing	NSA	ETL	BW	FOV	Matrix	Other	Parallel imaging
	(ms)	(ms)	(mm)	(°)	(mm)]			(Hz/Pixel)	(mmxmm^2^)			(Grappa factor)
2D T2W FSE sag	4100	80	3	RFFA = 150	3.3 mm	2	17	355	220 × 220	224 × 320	DIXON	2
3D T2W SPACE sag	1500	101	0.8	140	–	2	66	324	250 × 250	602 × 640	–	2
2D T1W FSE sag	500	9	3	RFFA = 150	–	2	3	250	220 × 220	240 × 320	–	2
2D DWI trans (RESOLVE)	1088	60	4	180	4	–	–	920	220 × 220	160 × 160	b = 600 s/mm^2^	2
2D RG Phase-Contrast trans	26.3	9.88	8	20	–	2	–	745	140 × 140	192 × 192	VENC = 8–12	None

*T2W* T2-weighted, *T1W* T1-weighted, *TSE* turbo spin echo, *SPACE* sampling perfection with application optimized contrasts using different flip angle evolution, *DWI* diffusion weighted imaging, *RESOLVE* readout segmentation of long variable echo trains, *RG* retrospective gated, *sag* sagittal, *trans* transversal, *RFFA* refocusing flip angle, *VENC* velocity encoding factor, *TR* repetition time, *TE* echo time, *NSA* number of signal averaged, *ETL* echo train length (turbo factor), *BW* bandwidth, *FOW* field of view

### Analysis of magnetic resonance imaging

#### Morphology

Evaluation of the cervical spine morphology was conducted using the MRI images including the following variables; kyphosis, tonsillar ectopia, lateral atlas displacement, alar ligament signal changes, transverse ligament signal changes, lateral joint degeneration C0/C1, lateral joint degeneration C1/C2, reduced disc height, abnormal disc contour, Modic changes (type 1, 2 and mixed type 1 and 2), uncovertebral joint degeneration, facet joint degeneration, neural foraminal stenosis, spinal canal stenosis and vertebral artery loop. Tonsillar ectopia was measured using the sagittal midline image, with the McRaes line (running from basion to opisthion) as reference. An extension of the cerebellar tonsils of more than 3 mm below this line was considered a positive finding. The lateral atlas displacement was evaluated on the coronal images. Any lateral displacement of atlas over axis of more than 2 mm was considered a positive finding. The researcher responsible for assessing the morphology had extensive experience in spinal MRI research and reading and was blinded according to patient group.

#### Diffusion weighted imaging

**Fig. 1:**
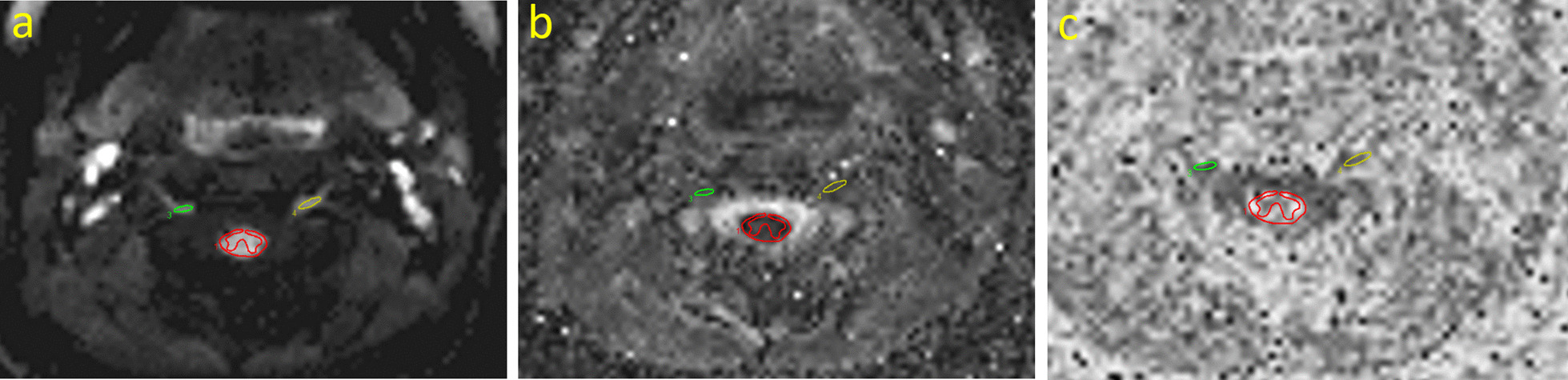
Diffusion MRI examples. *Legend* **a** trace image, **b** ADC and **c** FA images. ROI’s were drawn on the trace images and copied to the ADC and FA maps

The DWI data was analysed using the Spinal Cord Toolbox (SCT) [35]. The raw diffusion images were motion corrected and the apparent diffusion coefficient (ADC) and fractional anisotropy (FA) were calculated by solving the eigenvector equations. The values were assessed in five cervical spine nerve roots from C2 to C6 bilaterally. Each nerve root was marked on the average diffusion weighted images, at its most visible location on one to three axial slices on each side. The marked regions were copied to the ADC and FA images and mean values were calculated for analysis. Each region of interest (ROI) typically included 4–8 pixels. With approximately two slices per nerve root, eight to 16 pixels were used for the analysis of each nerve root. The spinal cord was marked on five axial slices from C2 to C6 and ADC and FA values were retrieved (Fig. 1). Care was taken to include white matter only. The white matter was manually segmented as we did not acquire T2*-weighted images which was a prerequisite for optimal function of the automatic segmentation function.

#### Cerebrospinal fluid flow

**Fig. 2:**
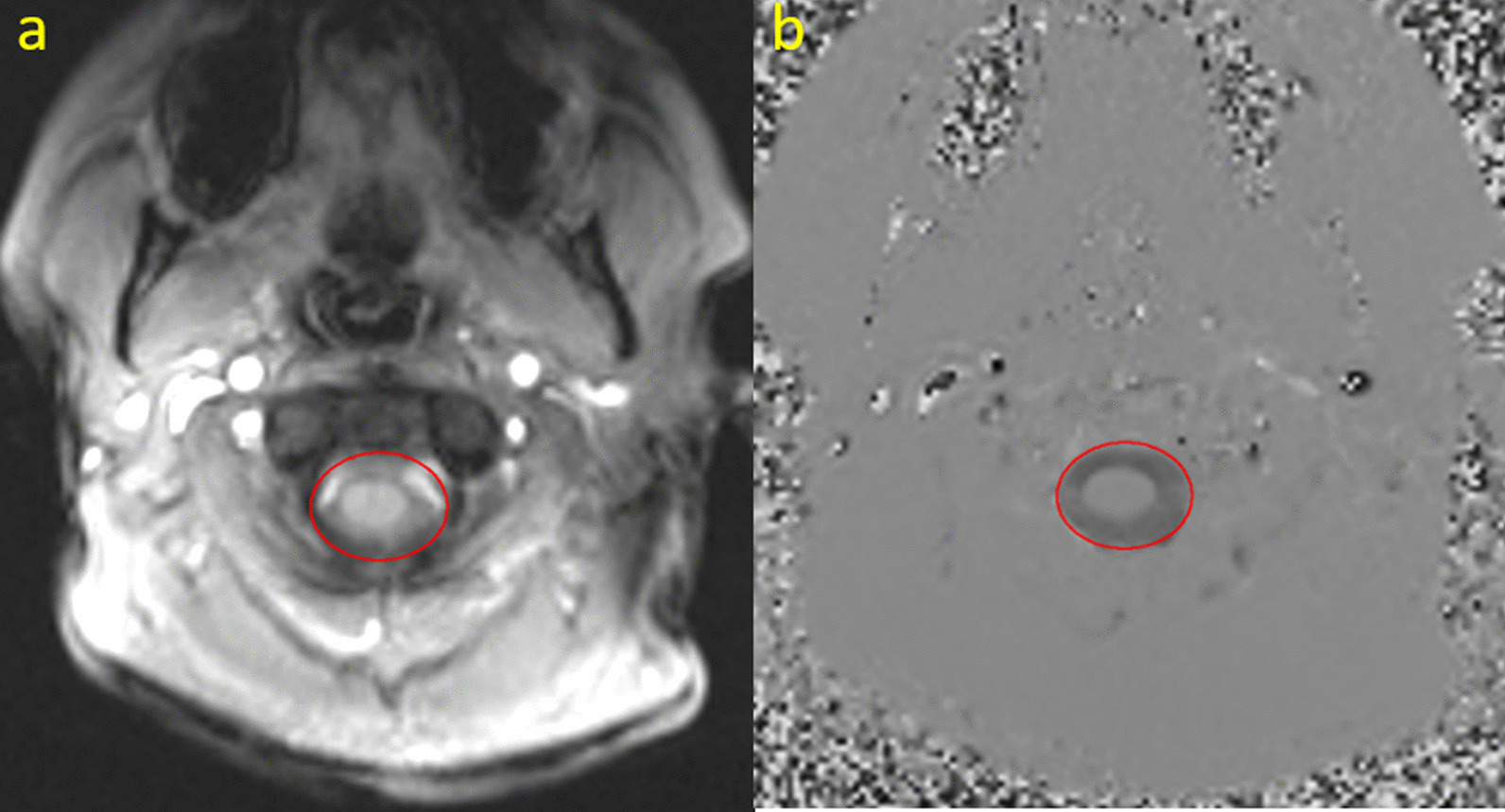
Phase contrast MRI example. *Legend* **a** anatomical image, **b** velocity image. ROI’s were drawn to include the spinal subarachnoid space and the spinal cord, while the velocities in the spinal cord were zero

The acquired PC-MRI data were used for measurements of CSF flow in 10 cases and 10 controls. ROI's were carefully positioned surrounding the spinal fluid (Fig. 2) and adjusted for all cardiac cycles. Volume flow curves were generated using the velocity images. Afterwards, the data were corrected for offset errors by assuming no net flow, i.e. the mean net flow was calculated and subtracted from the data. Based on the offset corrected flow curves, a number of parameters were calculated. These included mean positive and negative flow (ml/s), peak positive and negative flow values (ml/s) and total flow volume (gross forward and backward flow volume in ml) as described by Sartoretti et al. [28]. Furthermore, percentage of positive and negative velocity (%) and percentage time for maximum positive and negative flow (%) were also calculated. The former describes the percentage of positive and negative sample points while the latter describes at what percentage points during the flow cycle hold the maximum positive and maximum negative flow. All CSF flow analyses were performed using a post-processing software named SisWin, which is developed by one of the co-authors.

### Statistics

Data was collected from May 2019 to July 2020. Categorical data, e.g. MRI based morphology, were compared using Fisher’s exact test. For continuous data, e.g. cervical range of motion, means were compared between groups for the analysis of variance. ADC and CSF values were analysed using Mann Whitney U test. The statistical significance level was *p* < 0.05. Statistical analysis was performed using Stata^©^ 13.1 (StataCorp LP, College Station, USA) or Microsoft Excel 2019 with the statistical add-in tool pack Analyse-It® (Version 4.65.3, Analyse-it Software Ltd, Leeds, England).

## Results

A total of 20 chronic whiplash patients (cases) [11 women, range 20.4–50.7 years, 38.2 ± 9.7 years (mean ± SD)] and nine men, range 28.9–52.0 years, 37.3 ± 8.9 years) and 18 healthy subjects (controls) (ten women, range 23.7–57.1 years, 36.2 ± 7.4 years and eight men, range 23.4–49.7 years, 33.8 ± 10 years), were included in the study. Both groups were matched by gender and age (difference women: 0.3–4.4 years, difference men: 1.9–5.2 years).

### Questionnaires

#### Visual analogue scale—acute and current neck pain and headache

All 20 cases reported a VAS score for the acute onset of neck pain with a mean of 77.7 (SD 22.1, range 29–100), and nineteen cases reported a VAS score for current neck pain with a mean of 61.1 (SD 16.6, range 25–85) within the last 24 h. Nineteen cases reported a VAS score for the acute onset of headache with a mean of 78.3 (SD 18.9, range 34–100), and 17 cases reported a VAS score for current headache with a mean of 59.4 (SD 17.3, range 24–81) within the last 24 h. In addition, one control subject reported mild neck pain within the last 24 h on the day of examination and was omitted from the analysis of current neck pain. Similarly, another control subject reported mild headache within the last 24 h and was omitted from the analysis of current headache.

#### Reported non-painful neurological symptoms

The cases reported significantly more often non-painful neurological symptoms including dizziness, nausea, confusion, hypersensitivity to sound and light, visual disturbances, balance problems, cognitive challenges and paresthesia. Tinnitus was also more common among the cases, however not statistically significant (Table 2).

**Table 2:** Reported non-painful neurological symptoms

Neurological symptoms	Number of positive subjects		*p* Value^a^
	Cases (n = 20)	Controls (n = 18)	
Dizziness	11	0	< 0.001
Nausea	7	0	0.009
Confusion	8	0	0.003
Tinnitus	5	1	0.184
Hypersensitive to sound	15	0	< 0.001
Hypersensitive to light	12	0	< 0.001
Visual disturbances	8	0	0.003
Balance problems	8	1	0.021
Cognitive challenges	15	0	< 0.001
Parasthesias	13	0	< 0.001

^a^Fisher's exact two-sided

#### The Neck Disability Index

All 38 subjects answered the NDI questionnaire. The mean values were 22.35 (SD 8.23, range 13–39) for cases and 0.66 (SD 1.14, range 0–4) for controls (*p* < 0.0001). Hence, none of the controls showed disability, i.e. all reported NDI < 5, whereas all the cases showed varying degrees of disability (i.e. four mild, nine moderate, five severe and two complete disability).

#### The Copenhagen Neck Function Disability Scale

All 20 cases answered the CNFDS questionnaire. The mean value of the 20 cases was 19.4 (SD 6.14, range 10–29). All cases had disability according to the CNFDS score (i.e. five mild-moderate, seven moderate, five moderate-severe and three severe disability). There were no gender differences (*p* = 0.8861). Furthermore, one control subject reported neck pain within the last 14 days, scoring 1/30 on the CNFDS (based on “disturbed night sleep due to neck pain once in a while”) and was omitted from the analysis of the CNFDS data.

### Clinical examination

#### Neurological and orthopaedic findings

Clinical examination of the subjects showed significant differences between cases and controls. In particular, there were significant differences in neurological testing of the upper extremity with altered sensibility, reduced reflexes and reduced muscle strength. Furthermore, there were more positive orthopaedic findings among the cases involving cervical compression, distraction and palpation testing (Table 3).

**Table 3:** Clinical examination findings

	Number of positive subjects		*p* Value^a^
	Cases (n = 20)	Controls (n = 18)	
*Neurological findings*			
Rombergs test	0	0	N.a
Normal gait	0	0	N.a
Tandem gait	2	0	0.488
Nose-finger test	0	0	N.a
Alternate hand rotation	1	0	1.000
Cranial Nerves	0	0	N.a
Strength UE	5	0	0.048
Sensibility UE	9	0	0.001
Reflexes UE	11	0	< 0.001
*Orthopaedic findings*			
Cervical compression	10	0	< 0.001
Cervical distraction	7	0	0.009
Cervical palpation	18	1	< 0.001

^a^Fisher's exact two-sided

#### Active cervical range of motion

The mean values of the total ACROM (sum of all six rotational directions of the three-dimensional Cartesian coordinate system) were significantly reduced to a mean of 222° ± 70° for cases compared to a mean of 310° ± 46° for controls (*p* < 0.001), equivalent to an overall reduction of 28.3% in case subjects compared to controls. There were no significant gender differences in the total ACROM (*p* = 0.1290). Comparison of each unique direction showed significant differences in all directions with lower ACROM in the cases (Table 4).

**Table 4:** Active cervical spine range of motion (degrees)

Direction	Cases (n = 20)	Controls (n = 18)	Difference		
	Mean ± SD	Mean ± SD	Percentage (%)	Mean	*p* Value^a^
Flexion	32.2 ± 13.73	50.83 ± 15.62	36.7	18.63	< 0.001
Extension	37.5 ± 18.77	52.81 ± 13.06	29.0	15.31	0.007
Right rotation	50.5 ± 13.04	65.74 ± 7.32	23.2	15.24	< 0.001
Left rotation	46.8 ± 14.90	67.37 ± 7.39	30.5	20.57	< 0.001
Right lateral flexion	27.58 ± 9.96	36.22 ± 9.71	23.9	8.64	0.010
Left lateral flexion	27.23 ± 10.56	36.54 ± 11.27	25.5	9.31	0.013
ACROM total	221.82 ± 69.79	309.52 ± 45.58	28.3	87.7	< 0.001

^a^Analysis of variance (ANOVA)

#### Upper limb tension test

A total of six unique ULTTs were performed for all subjects. There were significant differences between cases and controls in four of the six tests with cases being positive more often; left median nerve (*p* < 0.001), left ulnar nerve (*p* < 0.001), right ulnar nerve (*p* = 0.045) and left radial nerve (*p* = 0.009). Testing the right median nerve and the right radial nerve provided no statistically significant differences (*p* = 0.410 and *p* = 0.232, respectively). There were no significant differences between gender in any of the six tests.

#### Algometry

According to Method 1, there was a significant difference between cases and controls with cases showing a lower mean pain threshold score of 3.35 kg/cm^2^ (SD 2.22) versus controls 4.85 kg/cm^2^ (SD 1.23) (*p* = 0.0160). There was a similar significant difference between gender with females scoring a lower mean pain threshold value of 3.41 kg/cm^2^ (SD 1.46) versus males 4.87 kg/cm^2^ (SD 2.21) (*p* = 0.0192). According to Method 2, the analysis of the full 18 points algometry showed, that cases had a significantly higher number of positive TePs with a mean of 12.2 (SD 5.2) compared to 6.1 (SD 2.8) in the controls (*p* = 0.0001). There were no significant gender differences.

#### Clinical hypermobility

According to the Beighton scale, one case was hypermobile (scored 5/9) and three controls were hypermobile (two scored 4/9 and one scored 5/9). None of the subjects had a Beighton score above five. There were no significant differences between the cases and controls, nor males and females, when grouping using the clinical cut point of a score of four (i.e. below four is “normal mobility”, four and above suggest “hypermobility”). When analysed based on the Beighton mean scores there was a significantly higher degree of mobility among females compared to males (*p* < 0.01), however the mean values were clinically insignificant as they were below four (i.e. 1.76, SD 1.73). No significant differences were observed between case and control subjects.

### Magnetic resonance imaging

#### Morphological findings

The evaluation of the cervical spine morphology generally showed, that the unique variables were reported more often among cases as compared to controls. However, there were no statistically significant differences in any of the variables examined between the cases and controls (Table 5).

**Table 5:** MRI evaluation of the cervical spine morphology

	Cases (n = 20)	Controls (n = 18)	*p* Value^a^
Kyphosis	2 (10%)	0 (0%)	0.49
Tonsillar ectopia	0 (0%)	3 (17%)	0.10
*C0/C1 and C1/C2*			
Lateral atlas displacement (> 2 mm)	1 (5%)	2 (12%)^b^	0.58
Alar ligament signal changes	6 (30%)	2 (11%)	0.24
Transverse ligament signal changes	4 (20%)	1 (6%)	0.34
Lateral joint degeneration C0/C1	0 (0%)	0 (0%)	1.00
Lateral joint degeneration C1/C2	0 (0%)	0 (0%)	1.00
*C2/C3*—*C7/Th1*			
Reduced disc height	8 (40%)	3 (17%)	0.16
Abnormal disc contour	11 (55%)	8 (44%)	0.75
Modic changes type 1	2 (10%)	2 (11%)	1.00
Modic changes type 2	1 (5%)	1 (6%)	1.00
Modic changes mixed type 1 and 2	2 (10%)	0 (0%)	0.49
Uncovertebral joint degeneration	11 (55%)	7 (39%)	0.35
Facet joint degeneration	6 (30%)	4 (22%)	0.72
Neural foraminal stenosis	3 (15%)	0 (0%)	0.23
Spinal canal stenosis	1 (5%)	1 (6%)	1.00
Vertebral artery loop	1 (5%)	5 (28%)	0.08

^a^Fisher's exact test

^b^One patient excluded due to cervical rotation C1/C2

#### Diffusion weighted imaging findings

No significant differences were found between the groups for neither ADC nor FA values pertaining to the spinal cord and nerve roots (Table 6).

**Table 6:** Analysis of diffusion weighted images of the cervical spine

DWI values	Cases (n = 20)	Controls (n = 18)	*p* Value^a^
	Mean ± SD	Mean ± SD	
ADC, nerve roots	1.58 ± 0.14	1.55 ± 0.13	0.22
FA, nerve roots	0.44 ± 0.05	0.44 ± 0.08	0.56
ADC, spinal cord	1.26 ± 0.13	1.27 ± 0.25	0.64
FA, spinal cord	0.68 ± 0.04	0.70 ± 0.06	0.21

*DWI* diffusion weighted imaging, *ADC* apparent diffusion coefficient, *FA* fractional anisotropy

^a^Mann Whitney U test

#### Cerebrospinal fluid flow findings

**Fig. 3:**
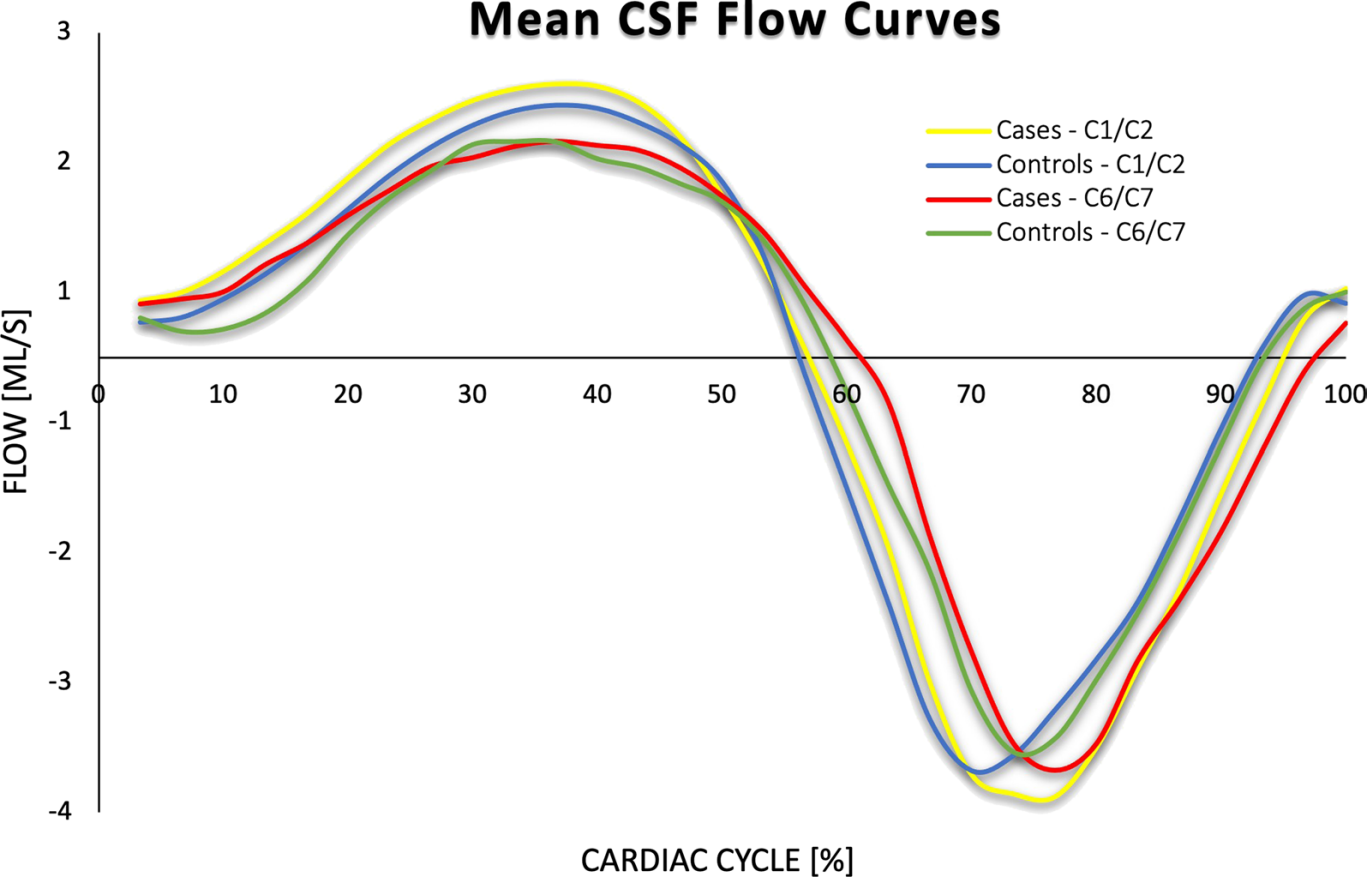
Mean cerebrospinal fluid flow curves for cases and controls at C1/C2 and C6/C7. *Legend* Mean flow curves obtained from offset corrected flow values and normalized to the cardiac cycle (%) at the C1/C2 and C6/C7 junction respectively

Cerebrospinal fluid flow data from 10 chronic whiplash patients (cases) [range 27.8–50.7 years, 36.7 ± 8.3 years (mean ± SD)] and 10 healthy subjects (controls) (range 25.3–43.8 years, 34.5 ± 7.0 years) were included in the study. Both groups were matched by age (difference: 0.1–8.9 years). The observed and calculated flow values of the CSF at C1/C2 and C6/C7 revealed no statistically significant differences between the groups (Table 7) (Fig. 3).

**Table 7:** Observed and calculated cerebrospinal fluid flow values at C1/C2 and C6/C7

MRI findings	C1/C2			C6/C7		
	Cases (n = 10)	Controls (n = 10)	*p* Value^a^	Cases (n = 10)	Controls (n = 10)	*p* Value^a^
	Mean ± SD	Mean ± SD		Mean ± SD	Mean ± SD	
Mean positive flow (ml/s)	1.40 ± 0.23	1.29 ± 0.81	0.44	1.16 ± 0.27	1.15 ± 0.40	0.68
Mean negative flow (ml/s)	− 2.56 ± 0.52	− 2.25 ± 0.76	0.35	− 2.10 ± 0.58	− 2.02 ± 0.87	0.74
Peak positive flow (ml/s)	2.24 ± 0.40	1.99 ± 0.42	0.25	7.84 ± 0.40	1.85 ± 0.52	1.00
Peak negative flow (ml/s)	− 4.23 ± 1.01	− 4.07 ± 1.04	0.44	− 3.62 ± 1.32	− 3.75 ± 1.22	0.68
± Stroke volume (ml)	26.97 ± 4.07	24.25 ± 5.91	0.39	22.19 ± 5.21	21.74 ± 7.70	0.74
% positive velocity	64.33 ± 4.17	62.67 ± 5.84	0.68	64.00 ± 5.62	63.00 ± 5.08	0.68
% Negative velocity	35.67 ± 4.17	37.33 ± 5.84	0.63	36.00 ± 5.62	37.00 ± 5.08	0.68
% Time for max positive flow	43.00 ± 8.53	39.00 ± 5.89	0.39	42.33 ± 19.75	48.67 ± 25.88	1.00
% Time for max negative flow	75.67 ± 7.17	74.67 ± 7.73	0.80	73.67 ± 4.29	74.33 ± 7.04	0.97

^a^Mann Whitney U test

## Discussion

This study of 20 chronic whiplash patients and 18 age and gender matched healthy controls investigated the feasibility of advanced MR-scanning techniques, including in particular DWI and CSF flow characteristics. The imaging procedures, including morphological evaluation, quantitative DWI and PC-MRI based CSF flow analysis of the cervical spine showed no significant differences between the whiplash patients and healthy controls.

### Clinical relevance

As expected, the cases and controls were significantly different based on their responses to the questionnaires and their clinical findings. The chronic whiplash patients in our study showed widespread chronic symptoms and generalized clinical findings, similar to previously investigated cohorts [3]. In particular, the reported symptoms and the aberrant findings on neurological examination and algometry support the presence of central hypersensitivity in these chronic pain patients. The clinical assessment tools utilized in this study were relevant in defining a study population of chronic whiplash patients. In fact, most of the tests are part of the DWGRASS stratification system, which enables baseline evaluation and prognostication at an early stage after whiplash injury [3, 4]. This evidence based risk assessment system of acute whiplash patients is based on ACROM, neck/head VAS and number of non-painful symptoms, and can after stratification, to a certain extent, predict outcome 12–14 years ahead [3]. In addition, we included two patient-reported outcome measures (PROMs), i.e. the NDI and CNFDS, which can be included in the management of patients with whiplash injury in support of the clinical observations made by the clinician [36]. Hence, for clinical settings we recommend an as early as possible objective clinical assessment of the whiplash injured patient, for the purpose of establishing an individual baseline. Future large-scaled practice-based studies implementing these methods may contribute to a better understanding of the course and management of patients with chronic whiplash injury.

### MRI cervical spine morphology

The findings of the MRI morphology were not significantly different between the cases and controls which is in agreement with the overall conclusions from a recent systematic review by Farrell et al. [15] and previous reports [37, 38]. We found a higher prevalence of alar and transverse ligament signal changes and reduced disc height among chronic whiplash cases, however these findings were not statistically significant. Our study did not reveal a higher prevalence of tonsillar ectopia in the chronic whiplash cases in contrast to previously reported [14]. Interestingly, the study by Freeman et al. [14] reported an even higher prevalence of tonsillar ectopia when examined during upright MRI in comparison to supine examination. These findings are relevant for future research studies of whiplash injured patients where there is access to an upright MRI facility. Overall, our findings support the current opinion that morphologic MRI of the cervical spine of chronic whiplash patients generally contributes minimally to the understanding and the clinical course of chronic whiplash injury [15, 37, 38].

### MRI DWI

The DWI protocol used in this study has previously showed good correlation between anatomical MRI and diffusion tensor imaging (DTI) in a post-mortem study [24]. Our study found no significant differences in FA or ADC values between the groups examined. This may in part be due to a potentially lower sensitivity of the clinical (in vivo) procedures compared to post-mortem, i.e. shorter scan time and higher risk of motion artifacts in the clinical study. Similar to our findings, a recent DTI study investigated the cervical spinal cord in 38 whiplash patients WAD grade I, II or III [16], demonstrating no significant differences between acute and chronic whiplash patients or at any group level on the DTI parameters (e.g. axial diffusivity and radial diffusivity). However, that study included only patients with loss of consciousness for a minimum of 30 min and/or brain injury among other inclusion criteria, which are the most common exclusion criteria for whiplash studies, therefore making comparison more difficult. Cohen-Adad et al. [39, 40] have recently published a consensus MRI protocol for state-of-the-art quantitative spinal cord MRI along with a post-processing pipeline based on the SCT. Their work supports the thesis that DWI may be beneficial for detecting spinal cord injuries. In our study, we used the SCT with the exception of the Magnetization Transfer sequence. Hence, including a Magnetization Transfer sequence for detection of white matter lesions in connection with spinal cord injury could have contributed further into detection of a difference between groups [39]. Future studies, potentially involving spinal cord injuries, should include the complete SCT recommendations.

### MRI CSF-flow

Our CSF flow investigation confirmed measurable motion at C2 and C7 in all subjects, in agreement with other recent studies [25, 26]. The findings were based on retrospective gated PC-MRI that was synchronized to the cardiac rhythm [27, 28]. Although our study identified CSF motion, under the assumption of no net flow, there were no significant differences between the groups examined. Hence, although the utilized PC-MRI protocol visualized motion in the CSF, it did not reveal any potential clinical relevance of this motion in our cohort of chronic whiplash patients. Disciplines with particular interest in CSF flow, e.g. Cranial Osteopathy and Craniosacral therapy, could consider utilizing the PC-MRI for the purpose of investigating this entity under different conditions.

### Limitations

This study included chronic whiplash patients recruited from a chiropractic clinic. Hence, the cases were a selected group of individuals, which could affect the external validity of the results. However, the included cases were relatively homogenous in their clinical appearance, and therefore we regarded the study population to be representative of chronic whiplash patients. Two control subjects reported mild current neck pain and mild headache respectively on the day of examination. As the symptoms were unspecific and short-lived and the subjects suffered no other symptoms they remained in the study. They were, however, excluded from the analysis of neck pain and headaches according to the VAS. We measured the CSF flow at an upper cervical (C2) and a lower cervical (C7) level for practical purposes. We are aware that measurements at different locations may provide other results. The applied DWI methods and analyses used in this manuscript have previously showed good correlation in a post-mortem study [24]. Applying them to this *in-vivo* study may however have led to less accurate estimates of the diffusion metrics. If so, this may in part explain why no statistical differences were observed between groups. In this respect, the null results could be related more to technical issues rather than a lack of difference between the groups. We are aware that our study in some aspects may suffer from underpowered analyses due to the relatively low number of participants.

### Feasibility of the study

This study investigated the feasibility of MRI techniques in a cohort of clinically well-described chronic whiplash patients and matched control subjects. Although we were unable to identify MRI-based biomarkers of chronicity in chronic whiplash patients, we showed that a multi-disciplinary clinical practice-based advanced imaging investigation is feasible. Financially, the costs were kept to a minimum by in-house funded access to MRI facilities and expertise as well as a symbolic reimbursement of transportations costs to the participating subjects. This study received external funding which supported the development of the MRI protocol. The authors received no financial compensation. Recruitment of subjects from the participating clinic and the consequent scheduling and execution of their clinical and MRI examination was managed as a collaboration of the participating chiropractic clinic and the Diagnostic Centre at Silkeborg Regional Hospital.

### Relevance for further research

This study identified no signs of a pathognomonic whiplash injury. Nonetheless, the negative findings in this study, i.e. no differences between groups with respect to DWI, CSF flow and structural changes on MRI are important as they may help guide future research in this field. In the context of the frequently reported painful and non-painful neurological symptoms and the clinical findings often observed among chronic whiplash patients (Tables 2, 3), the central nervous system including the brain, brainstem, cerebellum and nerve roots require further investigation. The use of DWI and CSF flow were feasible methods as shown in this study. However, a potential lack of sensitivity in small samples needs to be addressed by including larger sample sizes in future studies. Similarly, intra- and interobserver reliability of imaging findings should be included in future large scaled studies. Also, utilizing the SCT in full according to the MRI consensus protocol is recommendable [39].

## Conclusions

This clinical practice-based feasibility study of 20 chronic whiplash patients and 18 age and gender matched healthy controls examined the cervical spine morphology, DWI and CSF flow characteristics using advanced MRI techniques. Although no significant differences between the two groups could be established using the advanced MRI protocols, the PC-MRI sequences did visualize CSF flow within the spinal canal, which may be a relevant attribution to future cranio-cervical imaging studies. Our findings did not show that MRI‐based measures of morphology, spinal cord and nerve root diffusion and cerebrospinal fluid flow are sensitive biomarkers to distinguish between chronic whiplash patients and healthy controls. The detailed description of the chronic whiplash patients using readily available clinical tools may be of great relevance to the primary care practitioner. In the context of feasibility, clinical practice-based advanced imaging studies with a technical setup similar to the presented can be expected to have a high likelihood of successful completion.

## Data Availability

The datasets analysed during the current study are available from the corresponding author on reasonable request.
